# Objectives, design and main findings until 2020 from the Rotterdam Study

**DOI:** 10.1007/s10654-020-00640-5

**Published:** 2020-05-04

**Authors:** M. Arfan Ikram, Guy Brusselle, Mohsen Ghanbari, André Goedegebure, M. Kamran Ikram, Maryam Kavousi, Brenda C. T. Kieboom, Caroline C. W. Klaver, Robert J. de Knegt, Annemarie I. Luik, Tamar E. C. Nijsten, Robin P. Peeters, Frank J. A. van Rooij, Bruno H. Stricker, André G. Uitterlinden, Meike W. Vernooij, Trudy Voortman

**Affiliations:** 1 Department of Epidemiology, Erasmus University Medical Center, PO Box 2040, 3000 CA Rotterdam, The Netherlands; 2 Department of Respiratory Medicine, Ghent University Hospital, Ghent, Belgium; 3 Department of Otorhinolaryngology, Erasmus University Medical Center, Rotterdam, The Netherlands; 4 Department of Neurology, Erasmus University Medical Center, Rotterdam, The Netherlands; 5 Department of Ophthalmology, Erasmus University Medical Center, Rotterdam, The Netherlands; 6 Department of Gastroenterology, Erasmus University Medical Center, Rotterdam, The Netherlands; 7 Department of Internal Medicine, Erasmus University Medical Center, Rotterdam, The Netherlands; 8 Department of Dermatology, Erasmus University Medical Center, Rotterdam, The Netherlands; 9 Department of Radiology and Nuclear Medicine, Erasmus University Medical Center, Rotterdam, The Netherlands

**Keywords:** Biomarkers, Cancer and related diseases, Cardiovascular diseases, Cohort study, Dermatological diseases, Endocrine diseases, Epidemiologic methods, Genetic and molecular epidemiology, Nutrition and lifestyle epidemiology, Liver diseases, Neurological diseases, Oncology, Ophthalmic diseases, Otolaryngological diseases, Pharmacoepidemiology, Population imaging, Renal diseases, Psychiatric diseases, Respiratory diseases

## Abstract

The Rotterdam Study is an ongoing prospective cohort study that started in 1990 in the city of Rotterdam, The Netherlands. The study aims to unravel etiology, preclinical course, natural history and potential targets for intervention for chronic diseases in mid-life and late-life. The study focuses on cardiovascular, endocrine, hepatic, neurological, ophthalmic, psychiatric, dermatological, otolaryngological, locomotor, and respiratory diseases. As of 2008, 14,926 subjects aged 45 years or over comprise the Rotterdam Study cohort. Since 2016, the cohort is being expanded by persons aged 40 years and over. The findings of the Rotterdam Study have been presented in over 1700 research articles and reports. This article provides an update on the rationale and design of the study. It also presents a summary of the major findings from the preceding 3 years and outlines developments for the coming period.

## Introduction

The Rotterdam Study was designed in the mid-1980s as a response to the demographic changes worldwide that were leading to an increase of the proportion of elderly people [1]. It was clear that this would result in a dramatic increase in the number of persons living with chronic diseases, especially those with multi-morbidity, as most diseases cluster at the end of life. In order to discover the causes of diseases and thereby identify potential targets for preventive interventions one would have to study risk factors of those diseases [2]. A major approach to finding causes is the prospective follow-up study, which had proven highly effective in finding causes of heart disease and cancer.

## The design of the Rotterdam Study

**Fig. 1:**
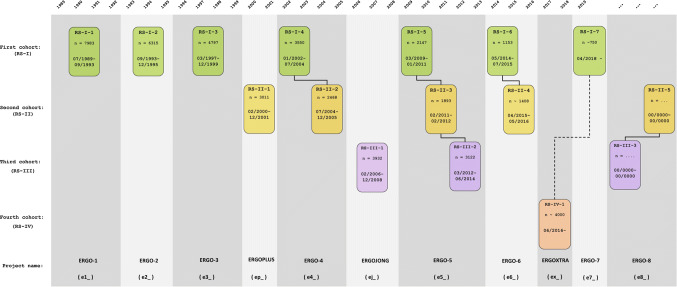
Diagram of examination cycles of the Rotterdam Study (RS). RS-I-1 refers to the baseline examination of the original cohort (pilot phase 07/1989–12/1989; cohort recruitment 01/1990–09/1993). RS-I- 2, RS-I-3, RS-I-4, RS-I-5, RS-I-6, and RS-I-7 refer to re-examinations of the original cohort members. RS-II-1 refers to the extension of the cohort with persons from the study district that had become 55 years since the start of the study or those of 55 years or over that migrated into the study district. RS-II-2, RS-II-3, and RS-II-4 refer to re-examinations of the extension cohort. RS-III-1 refers to the baseline examination of all persons aged 45 years and over living in the study district that had not been examined already (i.e., mainly comprising those aged 45–60 years). RS-III-2 refers to the first re-examination of this third cohort. Examination RS-I-4 and RS-II-2 were conducted as one project and feature an identical research program. Similarly, examinations RS-I-5, RS-II-3, and RS-III-2 share the same program items. Also, examinations RS-I-6 and RS-II-4 are conducted as one project. RS-IV-1 refers to the baseline visit of the fourth cohort, established in 2016. Re-examinations RS-II-5 and RS-III-3 for the second and third cohort will start early 2020

The Rotterdam Study was designed as a prospective cohort study, initially comprising 7983 persons living in the well-defined Ommoord district in the city of Rotterdam in The Netherlands (78% of 10,215 invitees). They were all 55 years of age or over and the oldest participant at the start was 106 years [3]. There were no prespecified exclusion criteria, meaning that all persons older than 55 years of age living in the area were invited to participate. The study started with a pilot phase in the second half of 1989. From January 1990 onwards participants were recruited for the Rotterdam Study. Figure 1 gives a diagram of the various cycles in the study.

In 2000, 3011 participants (out of 4472 invitees) who had become 55 years of age or moved into the study district since the start of the study were added to the cohort.

In 2006, a further extension of the cohort was initiated in which 3932 subjects were included, aged 45–54 years, out of 6057 invited, living in the Ommoord district.

By the end of 2008, the Rotterdam Study therefore comprised 14,926 subjects aged 45 years or over [4, 5]. The overall response figure for all three cycles at baseline was 72.0% (14, 926 out of 20, 744).

In summer of 2016, the recruitment of another extension started that targeted participants aged 40 years and over. The establishment of this extension is expected to be completed by early 2020 and to yield around 3000 new participants.

The participants were all extensively examined at study entry (i.e. baseline) and subsequent follow-up visits that take place every 3 to 6 years. They were interviewed at home (2 h) and then underwent an extensive set of examinations (a total of 5 h) in a specially built research facility in the centre of the district. These examinations focused on possible causes of invalidating diseases in the elderly in a clinically state-of-the-art manner, as far as the circumstances allowed. The emphasis was put on imaging (of heart, blood vessels, eyes, skeleton and later brain) and on collecting biospecimens that enabled further in-depth molecular and genetic analyses.

There were follow-up visits with re-exminations from 1990 to 1993, from 1993 to 1995, from 1997 to 1999, from 2000 to 2001, from 2002 to 2004, from 2004 to 2005, from 2006 to 2008, from 2009 to 2011, from 2011 to 2012, from 2012 to 2014, from 2014 to 2015, and from 2015 to 2016. In summer 2016 the aforementioned fourth cohort was established and underwent its first visit in the following years. The age range for this new cohort is predominantly 40–55 years. From 2018–2019 the first cohort was re-examined for the seventh time. Re-examinations for the second and third cohort will commence early 2020.

The participants in the Rotterdam Study are followed for a variety of diseases that are frequent in the elderly, which include but are not exclusive to coronary heart disease, heart failure and stroke, Parkinson disease, Alzheimer disease and other dementias, depression and anxiety disorders, macular degeneration and glaucoma, COPD, emphysema, liver diseases, diabetes mellitus, osteoporosis, dermatological diseases and cancer. In addition to the in-person examinations, the follow-up for these outcomes takes place via automated coupling of the study database with medical records from the general practitioners, who serve as gatekeepers to the Dutch health care system and therefore receive all relevant medical information from all caregivers of their patients.

The Rotterdam Study has been approved by the Medical Ethics Committee of the Erasmus MC (registration number MEC 02.1015) and by the Dutch Ministry of Health, Welfare and Sport (Population Screening Act WBO, license number 1071272-159521-PG). The Rotterdam Study Personal Registration Data collection is filed with the Erasmus MC Data Protection Officer under registration number EMC1712001. The Rotterdam Study has been entered into the Netherlands National Trial Register (NTR; www.trialregister.nl) and into the WHO International Clinical Trials Registry Platform (ICTRP; www.who.int/ictrp/network/primary/en/) under shared catalogue number NTR6831. All participants provided written informed consent to participate in the study and to have their information obtained from treating physicians.

For recent relevant EJE references see [6–29].

## Cancer and related diseases

### Overall aim and focus area

The age-adjusted incidence of many common cancers has increased in European populations over the past two decades. Moreover, cancer has taken over the role of most important cause of death in many developed countries. Therefore, more research with regard to cancer is necessary, not only to investigate its risk factors but also its treatment and determinants of survival. More and more, cancer is becoming a chronic disease which has an important place in a community-dwelling population of middle-aged and older individuals such as the Rotterdam Study. In the Rotterdam Study [RS], cancers are analysed as a clinical endpoint but also as a determinant or co-factor of other clinical endpoints.

### Key methods and data collection

Within the RS, cancer cases are registered via continuous follow-up of medical records from general practitioners. Furthermore, linkage with the Dutch Hospital Data (LMR) was established around 1998. The Dutch Hospital Data is a register which captures the main discharge diagnosis for all nationwide hospital admissions. Second, the Rotterdam Study was linked to PALGA, a local registry of histo- and cytopathology which captures all pathology reports in the region of the Rotterdam Study. In 2018, a linkage to the Netherlands Comprehensive Cancer Organisation (NKR) was established which will be updated every four years. The NKR is a nationwide registry with information on cancer diagnoses since 1989. This linkage gives additional information on first initiated treatment after diagnosis.

All potential cancer diagnoses are scrutinized on the basis of all available medical information, and independently classified by two physicians. Classification is according to the International Classification of Diseases and Related Health Problems, 10th revision (ICD-10) and the International Classification of Primary Care, 2nd edition (ICPC-2). The level of certainty of the diagnosis is established as: certain (pathology confirmed), probable (clinical diagnosis based on e.g. a mass on radiologic examination and/or biomarkers) or possible (e.g. an uncircumscribed mass by physical examination or a clinical presentation with painless jaundice and weight loss). The date of cancer diagnosis is registered as the date of the pathology report, or the date of the hospital admission if no pathology report was available. In case of disagreement, consensus is sought through consultation of a specialist in internal medicine.

### Main findings in the last 3 years

That population-based studies may be complementary to cancer registries follows from the underestimation of some cancers—such as pancreatic cancer—in registries [30]. We identified glutamine and histidine as biomarkers of potential biological interest to signal the presence of pancreatic cancer in an early stage [31]. In the Rotterdam Study, the focus of cancer research is on etiology, but also on prognosis. Furthermore, cancer may be a determinant or relevant co-factor in other RS-studies [32]. With regard to etiology, research has been done on diet [33–36] or lifestyle such as smoking [37, 38] as a risk factor, and laboratory assessments, for example, inflammatory markers in association with cancer [31, 39–41]. Furthermore, the association between cognition and cancer was studied [42, 43]. We have confirmed the positive association between a high baseline total cholesterol level and colorectal cancer (CRC) during follow-up, an association which was modified by dietary polyunsaturated fatty acids [35]. In individuals with a BMI below 25, a relatively high intake of glutamic acid was associated with a reduced risk of CRC [34]. Also adherence to 14 important items of the Dutch dietary guidelines was associated with a significantly lower risk of CRC [44]. In the Consortium on Health and Ageing (CHANCES), it was described that smoking, considerably advances, and cessation delays, the prognosis of CRC [37], but also of other cancers [38]. In addition, we found that dietary egg intake was associated with a higher risk of postmenopausal breast cancer [33] whereas a higher dietary zinc and iron intake were associated with a reduced risk of lung cancer [36]. With regard to laboratory assessments, we found that higher thyroid free T4 levels are significantly associated with an increased risk of any solid, lung, and breast cancer [45]. Also, inflammation, as measured by the systemic inflammation index [SII] was associated with a 30% higher risk of developing a solid cancer, making a high SII a strong and independent risk indicator for developing a solid cancer [40]. To enhance the usefulness of such markers, we assessed reference values for white blood cell based inflammatory markers [46].

### Future perspectives

In the near future, we will focus on 2 research topics. First, etiologically, we will investigate the role of drug use on the occurrence of cancer, especially use of long-term treatment such as calcium antagonists [47]. Furthermore, we will extend our investigation on the role of genetic determinants, diet and (inflammatory) biomarkers and the risk of cancer. Second, we will be elaborating on the potential prognostic effects of diet and inflammation after cancer diagnosis. The role of drug use as potential effect modifier on the survival of cancer will be investigated as well.

For additional EJE references please see [48–80].

## Cardiometabolic diseases

### Overall aim and focus areas

Research on the epidemiology of cardiometabolic disorders focuses on the etiology, prediction, and prognosis of cardiometabolic disorders including coronary heart disease (CHD), heart failure (HF), atrial fibrillation (AF), type 2 diabetes (T2D), and metabolic syndrome. This research line aims to provide sex- and gender-specific insights across the spectrum of cardiometabolic disorders.

### Key methods and data collection

#### Clinical follow-up

Information on clinical cardiometabolic outcomes is collected through an automated follow-up system which involves linkage of the study base to digital medical records from general practitioners in the study area and subsequent collection of letters of medical specialists and discharge reports in case of hospitalization. Clinical cardiometabolic outcomes are adjudicated according to established guideline-based definitions by study physicians and medical specialists [81].

#### Non-invasive measures of atherosclerosis

At baseline and follow-up examinations, ultrasonographic assessments of carotid intima-media thickness (cIMT) and plaques, measurements of carotid–femoral pulse wave velocity (PWV), ankle-brachial index, abdominal aortic calcification (X-rays of the lumbar spine), thoracic aortic diameters (ultrasound), echocardiographic measurements of structural and functional left and right heart parameters, and resting electrocardiogram are performed. Calcification in the coronary arteries, aortic arch, intra- and extra-cranial carotid arteries were assessed using CT. In case of carotid wall thickening on ultrasound, carotid plaque components were assessed using MRI.

Among 2000 participants with available EBT and carotid ultrasound, both proton Nuclear Magnetic Resonance (1H NMR) and Mass Spectrometry (MS) for metabolic profiling has been performed.

#### Sex- and gender-specific data

Questionnaire data to evaluate the impact of specific periods of potential vulnerability across a woman’s lifespan; menarche, pregnancy, reproductive lifespan characteristics and menopausal transition as well as measurements of sex hormone levels have been collected.

### Main results in the last 3 years

Atherosclerosis is a complex multifactorial condition involving multiple pathways influenced by both genetic and environmental factors. We investigated the role of inflammatory, oxidative stress, and hemostasis markers on cardiometabolic disorders. We found EN-RAGE as a novel inflammatory marker for pre-diabetes and for CHD [82, 83], IL17 for incident T2D and IL13 for pre-diabetes, incident T2D and insulin therapy [83]. We identified novel epigenetic correlates of circulating TNF-α and linked these loci to CHD risk [84]. We reported serum apoCIII levels, apoCIII-to-apoA1 ratio, visceral adiposity index, lipid accumulation product, the product of triacylglycerol and glucose to be associated with incident T2D, in particular in women [85, 86]. Mendelian randomization (MR) did not support the causal role of serum gamma-glutamyl transferase, as a marker of oxidative stress, on T2D risk [87]. ADAMTS13, a novel homeostatic factor, was an independent risk factor for incident T2D and CVD [88–91]. Our MR study did not support a large causal effect of fibrinogen on CHD [92].

Besides contribution to the global genetic discovery for CHD, HF, AF, and T2D [93–95], we also showed the biological interactions between genetic variants driving differential methylation and gene expression for T2D and highlighted the role of differential methylation in the crosstalk between adaptive immune system and glucose homeostasis [96]. We provided insights into potential biological mechanisms connecting tobacco smoking to excess risk of T2D and showed differential association of tobacco smoking with DNA methylation of the diabetes genes [97]. Among diabetic individuals, we identified 26 blood metabolomic measures to be associated with insufficient glycemic control, the strongest association was with glutamine [98]. Taking into account smoking behavior, multiple new loci for pulse pressure, mean arterial pressure, and blood pressure were identified, highlighting the importance of accounting for lifestyle factors and shared pathophysiology between cardiometabolic and addiction traits [99, 100]. We, however, did not find evidence of genetic interactions with body mass index on AF risk [101]. Despite similar lifetime risks of CVD at age 55 for men and women, men were more likely to develop CHD as a first event and women more likely to have stroke or HF [102]. Moreover, atherosclerosis (i.e. CAC) was present in approximately one-third of women categorized as being at low CVD risk based on the recent American guidelines [103]. CAC presence among low-risk women was associated with an increased risk of CVD [103]. Only 9.3% of men and 10.4% of women in the Rotterdam Study reached optimal cardiovascular health which was associated with sex steroids and sex hormone-binding globulin (SHBG) levels [104]. Total estradiol levels were also associated with presence of vulnerable carotid plaque and higher stroke risk in women [105]. Low levels of SHBG and high levels of total estradiol were associated with increased risk of T2D in women and higher serum dehydroepiandrosterone levels were associated with lower risk of T2D in both women and men [106, 107]. Among high-risk women with a history of polycystic ovary syndrome or premature ovarian insufficiency, we affirmed the potent impact of androgens on cardiometabolic features [108–110].

Early onset of natural menopause was an independent marker for T2D in women [111]. Women who experienced early menopause lived less long and spent fewer years without T2D than women who experienced normal or late menopause [112]. Moreover, genetic variants associated with earlier age at menopause increased the risk of CVD in women [113]. Furthermore, we showed that genetic variants associated with earlier age at natural menopause were associated with increased CVD risk in women, but not men, suggesting sex-specific genetic effects on CVD risk. Regarding the lifestyle factors, we further showed that smoking among women and metabolic factors (T2D and body mass index) among men had larger deleterious associations with longitudinal changes in left ventricular cardiac function [114].

We found lower levels of healthy ageing score (HAS) and sharper age-related decline in HAS among women compared to men [115]. Late first and last reproduction were associated with lower and a longer maternal lifespan, post-maternal fertile lifespan, and endogenous estrogen exposure were associated with higher all-cause mortality rates [116].

At age 55 years, the remaining lifetime risks for CHD, stroke, HF, AF, and T2D were 27.2%, 22.8%, 14.9%, 24.8%, and 28.1% for men and 16.9%, 29.8%, 17.5%, 22.9%, and 30.1% for women respectively [32, 102, 117, 118]. We further showed the implications of the major American and European guidelines at population level, quantifying the discrepant proportions of individuals eligible for statin treatment [119]. Among a range of newer markers, CAC and NT-ProBNP provide the largest increment in CVD risk prediction accuracy above the traditional risk factors [120]. We further examined the predictive ability of CAC versus age and showed CAC to be an alternative marker besides age to better discriminate between lower and higher CHD risk in older adults [121]. We took part in devising the updated global World Health Organization (WHO) algorithms for CVD risk estimation [122, 123]. To allow for routine use of risk charts in clinical practice, we showed that the non-laboratory-based models could predict CVD risk as accurately as the laboratory-based models [124]. Incorporating repeated measurements of blood pressure and cholesterol into CVD risk prediction models slightly improved risk predictions [125]. However, employing the novel deep learning algorithms using repeated-measures data led to greater discriminative accuracy for identifying people at high CVD risk compared to Cox regression approaches [126].

Although atherosclerosis is a systemic condition, we found persons with migraine, compared to those without, had less arterial calcification in the intracranial carotid artery, but not in other arterial beds [127]. We also showed that the association of impaired kidney function and larger volumes of arterial calcification was partly explained by cardiovascular risk factors. Arterial calcification did not mediate the association between kidney function and CVD beyond cardiovascular risk factors [128].

Higher cIMT, presence of carotid plaque, greater arterial stiffness, and larger volumes of epicardial fat were associated with higher AF incidence, indicating the role of atherosclerosis and arterial stiffness in AF pathogenesis [129, 130]. Carotid atherosclerosis was also associated with poorer hearing in older adults, suggesting that CVD prevention may also be beneficial for hearing loss in older adults [131]. Larger carotid artery diameter was also associated with risk of CVD, stroke, and mortality but not with CHD [132]. Among high-risk individuals, we showed baseline cIMT, but not cIMT change over time, to be associated with future CVD [133]. We further characterized vascular ageing by increasing arterial stiffness (PWV) and showed that participants with healthy vascular ageing were at the lowest end of the PWV distribution and had up to 14 years estimated younger biological (vascular) age than those with higher PWV values [134].

Active, high-dosage statin use beneficially influenced the composition of carotid atherosclerosis and shifted the composition from vulnerable plaque with a lipid core to more stable calcified plaque [135]. We showed both visual progression and regression of intra-plaque hemorrhage (IPH) volume during 17 months of follow-up [136], suggesting IPH as a dynamic process with potential for growth or resolution over time. Moreover, antithrombotic treatment related to a higher frequency of IPH in carotid plaques [137].

Our recent GWAs and colocalization analysis of cIMT and carotid plaque with vascular expression quantitative loci (cis-eQTLs) from relevant arterial wall and metabolic tissues implicated cIMT and carotid plaque loci in cardiovascular outcomes [138]. Our exome-wide association meta-analysis demonstrated that protein-coding variants in APOB and APOE associate with subclinical atherosclerosis. We showed the first significant association for APOE ε2 with multiple subclinical atherosclerosis traits across multiple ethnicities, as well as clinical CHD [139].

We characterized serum metabolic signatures associated with atherosclerosis in the coronary and carotid arteries and subsequently their association with incident CVD. The metabolites associated with atherosclerosis were largely consistent between the coronary (CAC) and carotid (cIMT) beds and predominantly tagged pathways that overlap with known cardiovascular risk factors [140]. However, we found differences in metabolic association patterns of intra- and extra-cranial carotid beds [141].

For additional EJE references please see [142–160].

## Dermatological diseases

### Overall aim and focus areas

The overall aim is to study common skin characteristics and diseases in a population based setting. The most important areas of research include skin cancer including basal and squamous cell carcinoma’s and melanoma; understanding the genetics of visible traits (e.g., skin aging, skin colour, hair colour and structure, eyebrow colour, facial shapes etc.) using facial digital 3D images; distribution of microbiome of the face and its determinants; phlebological outcomes including venous ultrasound of the lower extremities.

### Key methods and data collection

Participants are offered a full body skin examination by a dermatology trained physician. The focus of the clinical inspection is cutaneous (pre)malignancies, the presence of several inflammatory skin diseases such as atopic eczema, psoriasis and seborrheic eczema, and the presence of varicose veins. In addition, a 3D image of the face is collected for subsequent computer-vision based extraction of visible traits, the skin colour is assessed by spectroscopy, a skin swab of the nasal labial fold is taken, and a screening ultrasound of the venous system of the legs is performed.

### Main results in the last 3 years

Together with the Harvard cohorts we demonstrated for the first time that the genetic predisposition did not reveal new loci for developing multiple skin cancers [161], but based on clinical characteristics we developed a prognostic model [162]. In collaboration with other international consortia, the genetics of actinic keratosis, basal and squamous cell carcinoma, and melanoma have been further unraveled [163–166].

As member of different international consortia, we described many new and confirm previously known genes and performed genetic prediction studies for multiple visible traits such as male pattern baldness [167], perceived facial age [168], body height [169], hair color [170], hair structure [171], skin colour [172], eyebrow thickness [173], and eyebrow colour [174].

In collaboration with Unilever, several scientifically robust studies on different components of facial skin aging have been published in the last 3 years. First, we presented the largest population based study on the prevalence and determinants of facial skin aging [175]. Subsequently, Together with the longevity study from Leiden, we demonstrated that skin pigmentation genes were associated with wrinkling of the face [176]. Recently, we observed an association between a healthy diet and less facial wrinkling in women [177]. In a data driven analyses, we distinguished two different phenotypes of skin aging [178].

Seborrhoic dermatitis is a common skin condition (14% of participants of RS had physician diagnosed seborrheic dermatitis), but poorly understood. We demonstrated that men and especially those with lighter and dry skin were at risk of having seborrheic dermatitis [179]. A genetic analyses suggested that two loci play a role in the development of this disease [180].

### Future perspectives

The first batch of almost 1000 samples of the facial microbiome have been analysed. The distriubtion of the cutanoues micobiome in a large cohort will be studied as well as its relationship to other disease conditions. 3D facial images are continued to be collected to increase the power of future genetic studies.

For additional EJE references please see [181].

## Endocrine and metabolic diseases

### Overall aim and focus areas

The research line Internal Medicine focuses on diseases of internal organs, and how these diseases contribute to age related disorders such as cardiovascular disease and dementia. The main aim is to unravel mechanisms contributing to disease, thereby allowing new strategies for prevention, early detection and treatment.

Specific focus areas are hormone disorders (particularly thyroid disease) and the contribution of hormones to healthy aging and the development of age-related disease; kidney disease, prevention of renal insufficiency and the contribution of low kidney function to cerebrovascular diseases; immunity and the influence of the immune system on age-related disease; infections and how endogenous bacterial flora protect or contribute to disease development.

### Key methods and data collection

Serum and urinary analyses are the core business of the research line Internal Medicine:

- Hormone disorders: We are currently measuring a full profile of different thyroid hormone metabolites in a single run using LC–MS/MS technology, a novel and unique method developed in close collaboration with the Department of Clinical chemistry of Erasmus MC. Similarly, we have measured a full steroid profile using LC–MS/MS technology, as well as thyroid autoantibodies.
- Kidney disease: Next to the available kidney function measurements, serum creatinine, serum cystatin C and albuminuria, within the Rotterdam Study, we now also have repeated creatinine measurements available of participants through the STAR (laboratory and diagnostic center) which provides over 100,000 new measurements.
- Immunity: immunoglobulins were recently determined in ca 10,000 participants
- Infections: we are currently collecting nasal and pharyngeal swabs for microbiome studies

### Main results in the last 3 years

We have shown that an optimal thyroid function is essential for healthy aging. We have demonstrated that even in people without thyroid disease, high normal concentrations of thyroid hormones are related to an increased risk of sudden cardiac death [182], cardiovascular morbidity and mortality [183], dementia [184], frailty [185], type 2 diabetes [186] cancer risk [45], and a pro-coagulant state [187]. As a consequence, a high-normal thyroid state is associated with a decrease in life expectancy of 3.5 years for people with a high-normal thyroid function compared to low-normal [188]. Finally, we have identified multiple important novel genetic loci contributing to this difference in thyroid function [189]. These data together have identified thyroid hormone as a potential modifiable risk factor in the aging-related diseases, and have contributed importantly to the current treatment of patients with thyroid disorders [190, 191, 192].

In previous years we have focused on the relationship between low kidney function and brain health including cerebrovascular and degenerative disease. We have shown kidney function and kidney function decline to be association with stroke, but not for dementia [193]). Furthermore, we have been able to show that estimated glomerular filtration rates are independently associated with cerebral blood flow [194] and worse white matter microstructural integrity [195], which could represent a possible mechanism explaining the relation of low kidney function and brain diseases. On the other hand we have also sought possible new markers for kidney function decile and identified a von Willebrand factor: ADAMTS13 ratio (a marker for prothrombotic state) as possible risk factors for development of kidney disease [196].

### Future perspectives

We are currently investigating if specific thyroid hormone metabolites determined by LC–MS/MS can better delineate the role of thyroid hormone in the aging process and help to define the optimal health range for this hormone.

We have acquired information on end-stage kidney disease (dialysis or kidney transplantation) through collaboration with the Renine database, which will provide is with clinically relevant information on hard outcomes of kidney function. Furthermore, we are acquiring additional information on urinalysis performed in participants of the Rotterdam Study through general practitioners and laboratory and diagnostic centers.

For additional EJE references please see [197–208].

## Hepatogastrointestinal diseases

This research line is one of the youngest lines within the Rotterdam Study and focuses on major diseases of the liver, gut and stomach. Key focus areas include non-alcoholic fatty liver disease, non-alcoholic steatohepatitis, viral hepatitis, cancers of these organsystems, and liver dysfunction and fibrosis. The last few years, this research line has consolidated its efforts and focused primarily on continuity. The reader is therefore referred to the previous paper from the Rotterdam Study detailing the methods of this research line [209].

For additional EJE references please see [210, 211].

## Neurological diseases

### Overall aim and focus areas

Within the Rotterdam Study neuroepidemiologic research has primarily focused on the frequency, etiology and early detection of the following two major groups of age-related neurologic diseases: (1) neurovascular: stroke, including cerebral infarction, intracerebral hemorrhage, and transient ischemic and neurologic attacks and (2) neurodegenerative: dementia, including Alzheimer’s disease and Parkinson’s disease. As clinical symptoms in these diseases typically manifest themselves late in the disease course, our additional research focus is on pre-symptomatic brain pathology that can be assesses with non-invasive modalities, including magnetic resonance imaging (MRI), cognitive testing and gait assessments and more recently electromyography (EMG) for peripheral nerve assessment.

### Key methods and data collection

The most important source for incident cases of these neurologic diseases is through linkage of our database with files from the general practitioners, the municipality, nursing home physicians’ files and additional information (such as brain imaging reports) collected from hospital records [209]. In addition, participants, that visit the research center, undergo a screen for dementia with the Mini Mental State Examination (MMSE) and the Geriatric Mental Schedule (GMS), followed by an examination and informant interview with the Cambridge Examination for Mental Disorders of the Elderly (CAMDEX) in screen-positives (MMSE < 26 or GMS > 0), and subsequent neurological, neuropsychological and neuroimaging examinations [212, 213]. Furthermore, participants are screened for cardinal signs of parkinsonism (resting tremor, rigidity, bradykinesia, or impaired postural reflexes). Persons with at least one sign present are examined with the Unified Parkinson’s Disease Rating Scale and a further neurologic exam [214]. After thorough assessment of these sources, case reports are compiled, which are subsequently discussed by a panel led by an experienced neurologist [209, 212–217].

From August 2005 onwards (RS-II-2 and further), a dedicated 1.5 T scanner is operational in the research center of the Rotterdam Study, and brain imaging is performed in all study participants without contra-indications [218]. In addition to the MMSE, from the third examination round (RS-I-3) onwards, we added a 30 min test battery that was designed to assess executive function and memory function, and which includes a Stroop test, a Letter Digit Substitution Task, a Word Fluency Test, and a 15 words Word List Learning test [219]. This test battery was expanded from the fourth survey onwards (RS-I-4) to include motor function assessment using the Purdue Pegboard Test. Moreover, from 2009 onwards we expanded further by including the Design Orientation Test (DOT) and a modified version of the International Cooperative Ataxia Rating Scale (ICARS), which assess visuo-spatial orientation and ataxia respectively [220, 221]. Halfway through RS-III-1, we successfully implemented the assessment of gait in all participants using the GAITRite walkway (https://www.gaitrite.com/). Gait is assessed using a 5.79 m long walkway (GAITRite Platinum) with pressure sensors [222]. Finally, starting in January 2013, we have successfully implemented electromyography to assess polyneuropathy [223].

### Main findings in the last 3 years

In recent years, we have published data on the burden of common neurologic diseases in older adults in terms of life-time risks, including their co-occurrence and preventive potential. We found that one in two women and one in three men were diagnosed with dementia, stroke or parkinsonism during their lifetime [224, 225]. We further showed that strategies that could delay disease onset of all three diseases by 1–3 years, could potentially reduce these risks by 20–50%. These findings further highlight the importance of preventive measures that could reduce the burden of these common neurologic diseases in the elderly. For dementia, prevention trials that aim to delay cognitive decline are increasingly recruiting older individuals who are genetically predisposed to develop dementia. However, it remains unclear whether targeted health and lifestyle interventions can attenuate or even offset an increased genetic risk. Using long-term data on genetic and modifiable risk factors [226], we demonstrated that in individuals at low and intermediate genetic risk, favourable modifiable-risk profiles, including no current smoking, absence of depression, absence of diabetes, regular physical activity, absence of social isolation and adherence to a healthy diet, were related to a lower risk of dementia compared to unfavourable profiles. In contrast, these protective associations were not found in those at high genetic risk. These findings may aid in the design of future prevention trials. This was one of the first and largest studies to examine simultaneously the interplay between genetic and multiple lifestyle factors. Furthermore, in terms of gene–gene interaction, we found in another study that common variants with small individual effects jointly modify the risk and age of onset of dementia and Alzheimer’s disease, particularly in APOEe4 carriers [227].

In order to implement potential preventive measures, identification of individuals at high risk is essential. For existing prediction models, we showed high variability in discriminative ability for predicting dementia in the elderly highlighting the need for updated new models [228]. In a follow-up study, we developed and validated a prediction model to calculate 10-year risk of developing dementia in an aging population [229]. The basic model, which can be used in primary care setting, included information readily available from the anamnesis on age, sex, education, current smoking, history of diabetes, history of symptomatic stroke, depressive symptoms, parental history of dementia, presence of subjective memory complaints, need for assistance with finances or medication and systolic blood pressure available from the physical exam. Furthermore, an extended model was developed that could be used in a specialized memory clinic and that incorporated additionally cognitive testing, brain MRI markers and genetic data.

Finally, besides dementia [230–235], we are actively investigating the risk factors, burden and long-term prognosis of stroke [236–239], including transient ischemic and neurologic attacks, and parkinsonism (including Parkinson’s disease) [240–242], in the general elderly population. In recent years, we have also actively participated in several international genetic consortia to discover novel genetic loci for neurologic diseases and their preclinical endophenotypes [230, 243, 244, 245–247].

### Future perspectives

Traditionally, the focus within the neuro-epidemiologic research line has strongly been on dementia and stroke. In the coming years, we aim both to strengthen our research in the field of Parkinson’s disease and migraine, and extend to other neurologic diseases, such as epilepsy.

For additional EJE references please see [228, 237, 248–276].

## Ophthalmic diseases

### Overall aim and focus areas

Ophthalmic research in the Rotterdam Study focuses on occurrence, determinants, and predictors of common eye diseases which have a high risk of severe visual loss. Our main focus is on age-related macular degeneration (AMD), glaucoma, and myopia, and particularly in the last few years we investigated genetic risk variants and pathways. To this end, we connected with many other epidemiologic studies in all parts of the world and formed large international consortia.

### Key methods and data collection

We have not changed methodology after the 2018 update. In short, we perform an extensive eye examination at each round at the research center including best-corrected visual acuity (ETDRS), refractive error, Goldmann applanation tonometry, keratometry and ocular biometry (Lenstar, Haag-Streit), corneal topography (Pentacam; Oculus), and visual field testing (Frequency Doubling Technology C20-2, Zeiss Meditec). After pharmacological mydriasis, we make 35° color photographs of the macular area, and 20° simultaneous stereoscopic imaging of the optic disc and macular area using stereoscopic digital imaging (Topcon camera). We image retinal layers at the macula and optic disc with Fourier 3D Spectral domain optical coherence tomography (Topcon), and perform fundus autofluorescence, near infrared, and red-free measurements (Heidelberg). The classification of AMD, POAG, refractive error, and retinal vessel diameters remain unchanged.

### Main findings in the last 3 years

A European project focusing on AMD (EYE-RISK) was launched in 2015 and ended in 2019. For this project, we obtained crude data on AMD and all its determinants from 20 studies (E3 consortium), and established the largest AMD database in Europe consisting of 53.000 participants. The prevalence of late AMD stages in this database was 10% for persons 85 + years, and projections indicated that the number of patients with late AMD will almost double by 2040 [277]. High HDL cholesterol was significantly associated, which is surprising given its opposite relation to cardiovascular disease [278]. We identified an association with protein-altering variants in the COL8A1 gene with a whole exome platform [279]. A healthy diet was protective: persons following the so-called Mediterranean diet had a 41% reduction in risk of late AMD [280]. We also investigated determinants outside EYE-RISK. In the 3CC study, we found that the combination of genetic risk factors, early AMD phenotype, smoking, low intake of fish and lutein-zeaxanthin best predicted progression over 10 years [281]. Using our own Rotterdam cohort, we showed that a diet 200 g per day of vegetables, fruit two times per day, and fish two times per week reduced the risk of late AMD by half [282].

We continued our search for genes within the CREAM and 23andMe consortium (161 K persons), and identified 161 loci for refractive error. The genes suggest a light-dependent retina to sclera pathway in which all retinal cell types are involved [283]. We also found a close relation between refractive error, eye length, age, and visual loss: one in 3 persons with refractive error worse than -6 diopters (eye length 26 + mm) will become severely visually impaired, as will 95% of those with eye length 30 + mm (− 15D) [284].

For glaucoma, we also continued the genetic search in the IGGC consortium. We found additional genes for intraocular pressure (IOP), blood pressure traits, and POAG, and found a strong genetic relation between IOP and POAG [285]. We found no evidence for a common genetic background between POAG and myopia [286]. We did find an association with microRNAs [287]. We also focused on imaging, and found associations between a thinner retinal nerve fiber layer thickness and age, IOP, visual impairment, and history of systemic hypertension and stroke. A thicker nerve fiber layer was associated with smoking [288]. We linked image parameters from the retina to brain MRI images. Thinner upper layers of the retina were associated with gray and white matter changes particularly in the visual pathway [289]. We found no relation with migraine [290].

### Future perspectives

Our goal for the future is to link the genetic factors found for these important eye disorders to the presentation of the phenotype, and to the interaction with environmental factors. We particularly aim to assess how persons with a high genetic load can alter their lifestyle to diminish their lifetime risk. We will improve quantification of our disease outcomes with algorithms developed by artificial intelligence, which will help improve our predictions.

## Otolaryngological diseases

### Overall aim and focus areas

The otolaryngological research within the Rotterdam Study aims to gain insight in the etiology and impact of age-related hearing loss. Age-related hearing loss is a common disorder that deprives older people of key sensory input, with potentially severe consequences for social well-being and mental health [291, 292]. Our main areas of research are prevalence of age-related hearing loss; identification of determinants and risk factors of age-related hearing loss; associations of hearing loss with brain morphology and cognitive decline.

### Key methods and data collection

Hearing loss is assessed at both ears by performing pure-tone audiometry in a sound proof room. Hearing thresholds are determined with headphones at frequencies 0.25, 0.5, 1, 2, 4 and 8 kHz. To distinguish between cochlear and middle-ear pathology, also bone-conduction thresholds are measured at frequencies 0.5 and 4 kHz. Additionally, speech perception in noise is tested at the better ear, using a validated triplet digit test [293] with speech-shaped noise at a fixed presentation level of 65 dB SPL. The ability to understand speech in noise is a functional measure that includes both sensory and central aspects of the auditory system.

The general interview contains several general questions related to hearing problems. In case of hearing-aid use, the participant has to answer five additional questions of the International Outcome Inventory of Hearing Aids (IOIHA) [294]. In case of frequent tinnitus, ten additional questions of the Short Tinnitus Handicap Inventory (THIS) [295] are added.

### Main findings in the last 3 years

As expected, we found a high prevalence of hearing loss in our population [296]. About 30% of the population above 65 years were identified with a hearing loss greater than 35 dB HL at both ears, meeting the current Dutch indication criteria for hearing-aid use. A general association analysis revealed that hearing loss was independently associated with age, education, systolic blood pressure, diabetes mellitus, BMI, smoking and alcohol consumption [297]. Further exploration of the association between hearing and BMI showed a strong relationship for fat-related BMI, but no clear association with general diet quality [298]. Carotid atherosclerosis was identified as another potential risk factor for hearing loss [131], suggesting an important role of vascular mechanisms in the etiology of hearing loss.

Genetic susceptibility to age-related hearing loss has been investigated in a large meta-analysis within the international CHARGE consortium *(accepted for publication in Scientific Reports)*. Associations were found with 5 novel variants. In addition, several genes previously associated with age-related hearing loss were confirmed. Interestingly, different associations were found for low- and high-frequency hearing loss, confirming that different cochlear structures are involved in the etiology of age-related hearing loss.

As hearing loss may have a possible negative impact on cognitive function in an aging population, we studied the association between age-related hearing loss and brain morphology. Hearing loss was independently associated with a smaller brain volume, mainly driven by associations with white matter volumes [299].Further analyzes revealed additional associations between hearing and the level of organization of the white-matter microstructure [300]. Poorer hearing was associated with a poorer white-matter integrity.

### Future perspectives

We will continue our research in the current areas of interest. Additionally, future research will focus on longitudinal analyzes of hearing loss as these data have recently become available. Another new topic of research is tinnitus, which is closely related to hearing loss.

## Psychiatric diseases

### Overall aim and focus areas

The overall aim is increasing the understanding of etiology, course and effects of psychiatric symptoms and disorders in the general population across the life course, focusing on common psychiatric diseases and their symptoms, such as depression, anxiety, complicated grief and sleep disturbance. Over the last years our research particularly focused on (1) the etiology and interrelation between psychiatric disease symptoms across disorders and (2) the relation of psychiatric disease and its symptoms with physical and cognitive health.

### Key methods and data collection

Data collection on psychiatric phenotypes in the Rotterdam Study has been ongoing since 1993. One of our main phenotypes of interest, depression, was first measured with the Hamilton Depression Anxiety Scale (HADS-Depression subscale) and since 1997 with the Center of Epidemiology Scale-Depression (CES-D). Additionally, semi-structured clinical interviews have been used to obtain clinical diagnoses of depressive disorder (Schedules for Clinical Assessment in Neuropsychiatry, SCAN; since 2016 Lifetime Depression Assessment Self-report, LIDAS). Unique to the Rotterdam Study is the follow-up of medical records for depression diagnoses, currently being further expanded. Anxiety has been assessed with the Hamilton Depression Anxiety Scale (HADS-Anxiety subscale) and a slightly adapted Munich version of the Composite International Diagnostic Interview (CIDI). Follow up of medical records is also done for anxiety diagnoses. Sleep was measured subjectively and objectively in the Rotterdam Study. It is measured subjectively with the Pittsburgh Sleep Quality Index (PSQI) in every participant and additionally in subsamples with the Berlin Questionnaire (BQ), an adapted version of the Munich Chronotype Questionnaire (MCTQ), and a 7-day sleep diary. Objectively estimated sleep is repeatedly available by means of actigraphy, for which participants wear an accelerometer for 7 days and nights around their wrist; this is now part of routine data collection. Lastly, in 929 participants a 1-night polysomnography has been recorded. Additional current data collection focusses on complicated grief with (Inventory of Complicated Grief, ICG), aggression (Aggression Questionnaire, AQ), sexuality, social support, loneliness (UCLA Loneliness scale, 3-item version) and end of life decisions.

### Main results in the last 3 years

To further increase our understanding of the etiology of depression, we employed genetic and epigenetic approaches. Genetic analyses suggested new genes that might play a role in depression, for example RCL1 was identified as a novel candidate gene for depression [301] and 44 new independent loci associated with depression were found in a large GWAS [302]. In addition, methylation of 3 CpG sites associated with incident depressive symptoms, suggesting axon guidance may be a common disrupted pathway in depression [303]. Depressive symptoms have also been implicated on pathways to disease. High and increasing depressive symptom trajectories were associated with a higher mortality risk than stable low trajectories, while remitting trajectories were not associated with a higher risk [304]. Depressive symptoms were also found to be a mediator in the association of cardiometabolic dysregulations with cognitive decline [305]. Over the last three years, we also emphasized studying the role of sleep in brain health. Evidence for an association of subjectively rated sleep with white matter integrity is limited [306], but disturbed sleep as estimated with actigraphy was related to white matter integrity over time [307]. We also showed that while subjective sleep quality was not associated with the risk of dementia over a mean follow-up of 8.5 years [308], it was related to an increased risk of Parkinson’s Disease over a similar follow-up period [309]. To help disentangle mechanisms underlying subjectively and objectively estimated sleep and 24-h rhythm characteristics multiple new loci have been identified using a GWAS approach [310, 311]. Genetic analyses did not detect significant SNP heritability for morning and diurnal cortisol [312]. Cortisol levels were lowered in those with complicated grief versus those with no grief of normal grief [313] and experiencing complicated grief was also associated with a poor sleep quality cross-sectionally, albeit not longitudinally [314]. In contrast, prolonged grief was associated with cognitive decline over 7 years of follow-up [315].

### Future perspectives

Future work will remain its focus on common psychiatric disorders and symptoms, not only as separate disease entities but also taking a trans-diagnostic approach to assess interrelations and shared pathways. This approach will also be taken to assess the relation of psychiatric health with cognitive and physical health. Sleep will remain a focus point as pone potential pathway as it is associated with most mental health disorders. Lastly, social health and stress will be increasingly studied as important components of health as well.

For additional EJE references please see [316–324].

## Respiratory diseases

### Overall aim and focus areas

The respiratory epidemiology research group of the Rotterdam Study (RS) aims to determine the prevalence and incidence of respiratory symptoms (e.g. chronic cough), lung function impairment and common respiratory diseases such as asthma and chronic obstructive pulmonary disease (COPD) in middle-aged and older adults. In addition, we investigate risk factors—encompassing genetic susceptibility, environmental exposures and life style factors—for respiratory symptoms and diseases, calculate the lifetime risk and develop genetic risk scores. Lastly, we aim to elucidate the heterogeneity and the pathogenesis of asthma and COPD as well as acute exacerbations of these chronic airway diseases in order to delineate novel therapeutic targets. The overarching objective is to improve patient outcomes by discovering biomarkers for early diagnosis and identifying novel therapeutic targets.

### Key methods and data collection

In the Rotterdam study, we perform repetitive measurements of spirometry, offering the opportunity to investigate longitudinal trajectories of lung function over time. Additionally, we have information on respiratory diseases from medical records of all participants. A major asset of the Rotterdam Study is the multidisciplinary extensive characterization of the participants, the long-term longitudinal follow-up and the interdisciplinary collaboration to study multi-morbidity in older subjects.

The RS is a founding partner of the CADSET (Chronic Airway Diseases Early stratification) consortium, an European Respiratory Society (ERS) Clinical Research Collaboration studying the determinants and implications of different lung trajectories through life [325]. In collaboration with the CHARGE consortium, we have elucidated the genetic determinants of spirometric impairment, defined as either low lung volumes (Forced Vital Capacity [FVC]) or airflow limitation (decreased ratio of Forced Expiratory Volume in One second [FEV_1_] to FVC) [326–328].

### Main results in the last 3 years

We have determined the diffusing capacity of the lung measured by uptake of carbon monoxide (DLCO) in participants of the RS, determined its heritability and investigated the genetic determinants of lung diffusing capacity [329]. In a genome-wide association (GWA) study in collaboration with the Framingham Heart Study, we identified a genetic variant in *ADGRG6* which was significantly associated with DLCO per alveolar volume (DLCO/AV), an important measure of pulmonary gas exchange. Moreover, expression of *ADGRG6* was decreased in the lungs of subjects with decreased DLCO/AV and patients with COPD. Since ADGRG6 is a G protein coupled receptor (a drugable target), it might be an interesting therapeutic target for emphysema-predominant COPD patients.

Asthma is a heterogeneous disease affecting subjects at all ages. In the RS we have determined the prevalence of asthma in middle-aged and older subjects [330]; 3.6% of the approximately 15.000 participants (59% women, mean age 65 years) had physician-diagnosed asthma, with a higher prevalence in females (4.2%) than in males (2.8%). Subjects with asthma had a significantly higher prevalence of depression and obesity [330]. The RS has contributed to a large multi-ancestry GWA study of asthma, performed by the Transatlantic Asthma Genetics Consortium (TAGC), identifying five novel asthma risk loci [331].

We have shown that COPD is associated with an increased risk of peripheral artery disease [332], sudden cardiac death [333] and the development of atrial fibrillation [334]. COPD subjects with frequent exacerbations, with an enlarged left atrium on echocardiography or increased systemic inflammation had a significantly increased risk to develop atrial fibrillation [334]. Since atrial fibrillation is often asymptomatic and is an important cause of (embolic) stroke, this association between COPD—especially during or following acute exacerbations—and atrial fibrillation has implications for clinical practice. In a collaborative GWAS we identified 82 genetic loci significantly associated with COPD, of which 14 were shared with asthma or pulmonary fibrosis, confirming our previous observations of overlap between COPD loci and loci for lung function and pulmonary fibrosis [335]. Through epigenetic and transcriptomic studies, we demonstrated that genetic variants at chromosome 15q25.1 (encompassing the nicotinic acetylcholine receptor 3 [CHRNA3] gene and the iron-responsive element binding protein 2 [IREB2] gene) are differentially methylated in blood and differentially expressed in lung tissue of COPD cases and controls [336]. Similarly, we have elucidated the relation of the top COPD GWAS variant at chromosome 19q13.2 with DNA methylation and gene expression in blood and lung tissue [337].

### Future perspectives

The respiratory epidemiology research group aims to strengthen the epidemiologic and translational research within asthma and COPD, and to expand the spectrum of diseases investigated within the RS. First, asthma and COPD are heterogeneous diseases encompassing multiple clinical phenotypes and molecular endotypes with major differences in clinical presentation, etiology, natural history, prognosis and response to treatment. In the coming years we want to unravel further the pathogenesis, causes and mechanisms of asthma and COPD, both during stable disease and at acute exacerbations. Second, within the RS there are unique opportunities to investigate other respiratory diseases including interstitial lung diseases, sleep disordered breathing (obstructive sleep apnea syndrome), pulmonary hypertension [338], respiratory infections, chronic cough and lung cancer [36]. Indeed, chest CT scans have been performed in 2.500 participants; in 1.000 of these subjects chest CT imaging has been repeated after an interval of 10–12 years. Third, through linkage with pharmacy data, electronic medical records as well as cancer and mortality registries, the RS is ideally suited for pharmaco-epidemiologic studies. Lastly, using a systems biology approach, we aim to elucidate the pathogenic pathways of respiratory diseases by integrating multiple omics platforms (e.g. genomics, epigenomics, transcriptomics, proteomics and metabolomics) in clinically well phenotyped participants with long-term longitudinal follow-up.

For additional EJE references please see [339–345].

## Genetic and molecular epidemiology

### Overall aim and focus areas

Genetic epidemiology and molecular epidemiology are emerging innovative fields of research in which molecular, cellular and biochemical concepts and techniques are incorporated into computational models and epidemiological studies to identify determinants of human diseases. The team in these research lines focusses on bio-banking of the biological samples of participants of the Rotterdam Study, and investigation of molecular biological determinants of complex diseases. Bio-banking involves collecting, storing and managing the biological tissues of the Rotterdam Study participants at all follow-up measurements. This concerns mainly blood, urine, saliva, hair and faeces but with microbiome studies several other specimens are being collected (such as skin swaps, nose swaps, eye swaps, etc.). We have further stored peripheral blood mononuclear cells (PBMCs) for the isolation of induced pluripotent stem (iPS) cells. The main research focuses include identification of genetic predictors for disease and treatment response, and assessment of biological mechanisms underlying complex diseases using various biomaterials (e.g., DNA, RNA, proteins, metabolites, microbes) measured with novel high-throughput –omics technologies. The materials and data generated by this research line now sum up to ~ 3 × 10^12^ data-points, and are actively used by all research groups of the Rotterdam Study. An overview of all the “omics” datasets in the Rotterdam Study cohorts is given in Table 1.

**Table 1:** Overview of sample numbers with “omics” datasets across the three Rotterdam Study cohorts with the number and type of measurement for each omic method

Omics data type	Total	Data point	Number	RS-I	RS-II	RS-III	RS-IV
GWAS SNP data	11,502	SNPs	40,000,000	6291	2157	3054	Ongoing
Exome array	3183	SNPs	250,000	3183	–	–	–
Whole-exome Seq	3778	Variants	693,000	3778	–	–	–
Whole-genome Seq (WGS)	96	Variants	3,000,000	96	–	–	–
Genome-wide expression (array)	881	Genes	25,000	–	–	881	–
Genome-wide expression (RNA-Seq)	829	Read	18,000,000	27	504	276	–
Genome-wide DNA methylation	1600	CpGs	450,000	69	468	1003	–
Gone-wide microRNA profiling	2750	miRNAs	2083	1000	1000	–	750
Serum protein profile^a^	9820	Proteins	35	3812	2542	3466	-
Proteomics	3596	Proteins	92 + 92	–	–	3596	–
Metabolomics untargeted (NMR/UPLC/MS)	1826	Metabolites	4000	1826	–	–	–
Metabolomics targeted (Nightingale platform)	5381	Metabolites	228	2880	593	1788	–
Metabolomics targeted (Metabolon platform)	488	Metabolites	855	488	–	Ongoing	–
Gut Microbiome (16S rRNA)	2000	OTUs	500	–	–	2000	–
Mitochondrial DNA (PCR)	500	1	–	500	–	–	–
Telomer length (PCR)	1800	1	–	1800	–	–	–
Total ‘omic’ datapoints in RS: 46,434 × 62,425,546 = 2,898,667,802,964							

*SNP*, single-nucleotide polymorphism; CpG, a two-nucleotide position (C next to G on the same strand) of which the C can be methylated; OUT, operational taxonomic unit; RS-I, first cohort of the Rotterdam Study; RS-II, second cohort of the Rotterdam Study; RS-III; third cohort of the Rotterdam Study; RS-IV, fourth cohort of the Rotterdam Study

^a^Serum proteins profile include total estradiol, total testosterone, sex hormone-binding globulin, dehydroepiandrosterone, dehydroepiandrosterone sulfate, androstenedione, 17-hydroxyprogesterone, cortisol, corticosterone, 11-desoxycortisol, vitamin D, thyroid stimulating hormone, free T4, interleukins, C-reactive protein, Insulin-like growth factor 1, insulin, iron, ferritin, transferrin, fibrinogen, homocysteine, folic acid, riboflavine, pyridoxine, SAM/SAH ratio, cobalamine, Lp-PLA2, Fas/Fas-L, abeta42/40

### Key methods and data collection

At each examination at the research center, blood, serum, plasma (citrate, heparine), urine and saliva is collected, as well as EDTA tubes for DNA and PAXgene tubes for RNA isolation. Fasting blood samples are collected along with challenged samples as part of a glucose tolerance test. Saliva is collected before and after a dexamethasone-suppression test. Saliva is frozen at − 196 °C before and after the challenge, and stored at − 80 °C. To obtain serum and plasma, tubes are centrifuged according to a protocol standardising time and conditions from the drawing of blood to centrifugation. All samples including the full blood are snap-frozen at − 196 °C using liquid nitrogen and stored at − 80 °C. Overnight urine samples are collected at home, frozen at − 196 °C at the research centre and stored at − 80 °C.

DNA is isolated from whole blood at one laboratory at Erasmus MC by a manual salting-out protocol and is subsequently stored in Eppendorf tubes at -20 °C, and in later rounds in Matrix 2D-barcode tubes. A copy of the complete DNA collection of ~ 13,000 samples has been transferred to Matrix 2D-barcode tubes in 96-well format at another location. This copy has been subjected to normalization of DNA concentrations and made suitable for handling in 96- and 384-well micro-titer plates for subsequent downstream genomic analysis.

Starting with the RS-III round of data collection, blood drawing has also been taken place with PAXgene tubes, from which whole RNA is isolated and stored at − 80 °C. This is now ongoing for the whole study population following the cycles of visits to the research centre.

Similarly, with the RS-III round, collection of faeces material has been initiated for the intestinal microbiome analysis. A collection pot is distributed at the research centre visit which is to be used at home and then by postal mailed returned to Erasmus MC where DNA is isolated and stored at − 80 °C. This is now ongoing for the whole study population following the cycles of visits to the research centre.

For data management, an in-house customized sample-management system has been developed. The Rotterdam Study “omics” data (incl. GWAS data, RNA expression profiles, DNA methylation profiles, and Next-Generation Sequencing (NGS) data including whole-exome sequences, RNA-sequencing, and the microbiome 16S ribosomal RNA) are generated in the Human Genotyping Facility (HuGe-F) (www.glimdna.nl), while QC-ed and extracted data are stored and managed in the central data repository of the Rotterdam Study.

### Genotyping data

(A) The genome-wide association studies (GWAS) dataset of more than 12,000 DNA samples from the three Rotterdam Study cohorts consists of a) a small dataset of ~ 400 women with 500 K Affymetrix arrays (Nsp250 + Sty250; the so-called “pilot” dataset), and b) a large dataset of ~ 12,000 samples consisting of 550 K (RS-I, II; single + duo array format) and 610 K (RS-III; Quattro array format) Illumina array genotypes. In the pilot dataset also other array types have been run such as the Illumina Omniexpress 2.5 array, and the new Illumina GSA array and the Affymetrix PMRA array allowing for comparisons.
  The Illumina GWAS genotype datasets of the Rotterdam Study (RS) also form the basis to generate so-called “imputed” datasets derived thereof. In this process the genotypes of SNPs which have been genotyped in reference datasets (such as HapMap and 1000 Genomes), are being estimated for all Rotterdam Study samples using the basis Illumina 500 K SNP dataset configurations in each subject. With the advent of large reference datasets becoming available based on whole genome/exome NGS, imputation activities using the RS GWAS dataset will remain an active area of development. So far, the RS GWAS datasets have been imputed to HapMap version 2 and 3 (with ~ 2.5 million resulting imputed SNP genotypes obtained for the RS dataset), the 1000 Genomes (1 kg) dataset version Iv3 and IIIv5 (with ~ 30 and 50 million resulting SNP genotypes, respectively), the Genome of the Netherlands (GoNL), the UK10K whole genome sequencing dataset, and, more recently, the haplotype reference consortium (HRC) r1.1 dataset (~ 40 million SNPs). Especially the latter imputation uses as a reference up to 64,976 haplotypes allowing also the study of less frequent to rare variants and comprising 40 million SNPs, all with an estimated allele count greater than 5. Imputation of the RS GWAS datasets to the TOPMed reference panel is now ongoing with an expected number of 130 million variants.
(B) Whole-exome sequencing (WES) dataset in the RS is available for 2628 samples from RS-I as part of the NCHA sponsored project and were generated by the HuGe-F on the Illumina HiSeq2000 sequencing machines [346]. The samples for this experiment were selected to constitute a random sample from the RS-I dataset. Through a collaborative grant from the NIH Alzheimer initiative (ADSP), we have obtained an additional ~ 1200 samples with WES NGS data from RS-I generated at the Broad Institute, Boston, USA (of which 50 overlap with the NCHA WES dataset), so the net total number of samples with WES data is 3778. The RS WES dataset is now also part of the so-called commons dataset of the CHARGE consortium which has ~ 16,000 WES samples and 5000 WGS samples.
(C) Whole-genome sequencing (WGS) dataset consists of 100 samples also from the RS-I which were sequenced as part of the Genome of the Netherlands (GoNL) [347], with an average sequencing depth of 6 × and with improved phasing because of the trio-design.
(D) About 300 SNPs in several candidate genes have been individually measured over the past 15 years, (including genes such as ApoE, VDR, ESR1, fibrinogen, etc.). Additionally, for a subset of RS-I samples telomere length (~ 1800) and mitochondrial DNA content (~ 500) was measured. Also, the telomere and mitochondrial DNA will be measured (by RT-PCR) in the total DNA set of the Rotterdam Study, including DNA samples collected at follow-up visits. In addition, we will assess heteroplasmy of mitochondrial DNA in blood by NGS in a large subset of RS-I.

### Transcriptome data

With the availability of good RNA from Rotterdam Study participants, starting with the RS-III subjects, studies have been initiated analysing the expression pattern of a single gene across samples or of the complete RNA collection in a sample (expression profiling). An expression profiling dataset has now been generated for ± 900 samples of the RS-III dataset, using the Illumina Human HT-12 v4 array containing ~ 48,000 probes. Moreover, in a BBMRI-sponsored collaborative effort to create a large-scale data infrastructure to work on integrative omics studies in Dutch Biobanks, the HuGe-F has generated RNA sequencing profiles of in total ± 4000 individuals of six Dutch biobanks, including ± 900 samples from the RS-III-1, at a depth of 30 million paired end reads. Together there are a very rich RNA expression dataset of in total ± 1800 sample is now available in the RS-III-1. Yet while RNA expression is known to differ between tissues, so far we only have RNA isolated from whole blood as a tissue.

### Epigenome data

A. DNA methylation can regulate gene expression without altering the underlying DNA sequence and is now emerging as a promising molecular strategy for risk stratification for complex disease. In the same samples that have RNA expression profiles, see above, we have generated DNA methylation profiles of ~ 480,000 CpG sites across the human genome using the Illumina Infinium HumanMethylation450 array. As this same set of RS-III-1 subjects was also used for the RNA expression profiling, deep genomic studies can now take place in combination with the GWAS data and NGS data in these ~ 1600 subjects.

B. MicroRNAs (miRNAs) represent a class of small non-coding RNAs, which function as post-transcriptional regulators of gene expression via targeting the 3′-untranslated region of target transcripts. In a total number of 2750 RS participants plasma miRNA levels were determined. These include a random selection of 1000 participants from the fourth visit of RS-I (RS-I-4) and 1000 participants from the second visit of RS-II (RS-II-2), these visits were performed between 2002 and 2005 with follow-up visits every 4–5 years. In addition, 750 participants from the new visit of RS cohort (RS-IV-1) were selected. The miRNA levels was measured by the HTG EdgeSeq miRNA Whole Transcriptome Assay (WTA) (HTG Molecular Diagnostics, Tuscon, AZ, USA) and using the Illumina NextSeq 500 sequencer (Illumina, San Diego, CA, USA). The WTA measures the expression of 2083 human miRNAs, and the expression of 13 housekeeping genes. In this setting, quantification of miRNA expression was based on counts per million (CPM) and log2 transformation of CPM was used as standardization and adjustment for total reads within each sample.

### Metabolome data

Metabolomics is a rapidly growing field of study that endeavors to measure metabolites within a biological sample. New technologies in high-throughput metabolomics by mass spectrometry allow for an efficient profiling of metabolites in body fluids of numerous participants in large cohort studies. The metabolomic profiles can represent a momentaneous functional readout of the physiological state of the human body and may provide novel biomarkers for diseases. Multiple datasets have been created in the Rotterdam Study sub-cohorts that contain information on metabolomics.

(A) As part of the COMBI-BIO consortium, we used large-scale untargeted serum metabolic profiling by proton (1H) nuclear magnetic resonance (NMR) spectroscopy and UPLC Mass Spectrometry to characterize the metabolic signature of 1826 individuals from the third visit of RS-I (RS-I-3) in relation with vascular health and cardiovascular disease.
(B) High-throughput metabolomics measurements as a part of the Biobanking and BioMolecular resources Research Infrastructure the Netherlands (BBMRI-NL) initiative have been performed using plasma samples which were collected in EDTA coated tubes. Fasting samples from RS-I (n = 2880), RS-II (n = 663), and RS-III (n = 1838) cohorts have been specifically selected in order to maximize the analytical number of prospective gene expression and gut microbiome research in relation to metabolomics. The plasma samples analyzed by the biomarker platform of Nightingale Health (formerly known as Brainshake) using NMR technique. Spectra have been obtained from 600 to 500 MHz instruments, using three molecular windows, namely lipoproteins, lipids and low molecular weight compounds. The spectra were then de-convoluted by Nightingale’s proprietary bioinformatics software leading to quantification of absolute concentrations. The yielding biomarker data contains 228 measurements on apolipoproteins, lipoproteins sub-classes, amino acids, albumin, glucose, glycolysis metabolites, ketone bodies, glycoprotein, sphingolipid, phosphoglyceride, polyunsaturated fatty acids and cholesterols.
(C) Plasma metabolites of 488 participants of the RS-I-3 were measured by Metabolon Inc, in which ultra-high-performance liquid chromatography and gas chromatography coupled with tandem mass spectrometry were used to measure a large number and broad spectrum of molecules with a high degree of confidence [348]. Measuring plasma metabolites with this platform is now ongoing in ~ 1400 samples of RS-III-2.
(D) Urine metabolomics employing mass spectrometry, to perform both non-targeted urinary metabolomics as well as targeted quantification of eicosanoid metabolites in urine, is now ongoing in ~ 1500 samples of RS-II-3 and RS-II-2.

### Proteome data

Plasma levels of 92 inflammation-related proteins and 92 cardiometabolic-related proteions were measured recently in > 3000 participants from the RS-III-1 using two Olink's high-throughput assays (INFLAMMATION and Cardiometabolic) and are now ready to be used in the future studies together with other omics data in the Rotterdam Study.

### Microbiome data

The HuGe-F has optimized and applied stool/faeces collection protocols in a cohort setting, and used 16S sequencing protocols (NGS of the 16S rRNA v3/v4 area) to characterize the gut/intestinal microbiome. We have collected ~ 2000 stool samples in the RS-III-1 sub-cohort from which DNA has been isolated and which have been sequenced on 16S v3/v4 by NGS on Illumina MiSeq sequencing machines (Radjabzadeh, et al., https://doi.org/10.1038/s41598-020-57734-z). For other sources of microbiomes (eye, urine, mouth, skin, etc.) several pilot projects have shown their feasibility while sampling and sequencing protocols were optimized (e.g., for some microbiome body niches other 16S areas need to be sequenced). For all these other body niches larger sampling efforts are now ongoing in the ongoing collection rounds of the Rotterdam Study. These can be found under the description of the respective research lines.

### Main findings in the last 3 years

Rotterdam Study investigators are playing leading roles in several of the large international consortia focused on assessing the genetic determinants of complex diseases by prospective meta-analysis across many epidemiological cohorts, such as in CHARGE and ENGAGE, and in many disease/phenotype focused efforts such as ADSP, IGAP, PERADES, GIANT, GEFOS, REPROGEN, TREATOA, DIAGRAM. Since 2005 the genome-wide association study (GWAS) has changed the field of complex genetics, and identified a still growing list of thousands of common genetic variants contributing to disease risk. While this large scale global collaboration has originated from the GWAS era, similar consortia have been built around the genomics datasets with RNA expression profiles, DNA methylation profiles, and the NGS datasets on DNA, RNA and microbiomes, including the BBMRI-NL sponsored BIOS consortium and several CHARGE working groups. Especially, from the CHARGE consortium many important publications have emerged on a wide variety of phenotypes and diseases from all major research lines in the Rotterdam Study. They are discussed under the subheadings of each individual research line.

### Future perspectives

#### Incidental findings in whole-exome sequencing (WES) data

Based on the WES dataset and the exome chip dataset of the RS we have initiated a working group to look for so-called incidental findings which might be clinically relevant. This is done by determining presence of variants in particular sets of genes such as the list of 57 “actionable” genes as established by the American College of Medical Geneticists (AMCG). A first result showed that carriers of supposedly pathogenic mutations in the prion gene did not display an evident disease phenotype [349]. This phenomenon of reduced penetrance of supposedly pathogenic mutations is now under further investigation using the WES data of RS.

WES data was also used to investigate the association between all-cause mortality and carrier-status of somatic mutations in genes linked to clonal expansion of hematopoietic stem cells. We found that, unlike previous reports in predominantly middle-aged individuals, somatic mutations in genes linked to clonal expansion of hematopoietic stem cells do not compromise the 8- to 10-year survival in the oldest old [350].

#### Genetic risk assessment

Due to rapid progress in the outcomes of the many GWAS studies more and more, so-called polygenic risk scores (PRS) can now be calculated for numerous diseases as well as risk factors of diseases involving hundreds of SNPs per disease-specific PRS. In addition, the newer SNP arrays (such as GSA from Illumina, and PMRA from Affymetrix) also include many clinically relevant genetic markers in their content, such as pharmaco-genetic markers, and HLA. These developments have led to the formation of a new working group in CHARGE (genetic risk assessment) to further investigate the opportunities and limitations of such PRS and clinical variants in the setting of cohort studies, where return of results to participants is one aspect of consideration. It has also led to pilot studies to investigate the opportunities and limitations of applying such arrays in the clinical setting of (academic) hospitals.

#### Integrative omics and systems epidemiology

Within the Rotterdam Study sub-cohorts, various omics datasets (incl. genomics, epi-genomics, transcriptomics, proteomics, metabolomics, and microbiome) have been generated. Integration of these population-based omics data with the state-of-the-art molecular and cellular model systems would help true biological insight into mechanisms behind complex diseases.

The epigenetic and transcriptomic data have increasingly been explored for associations with disease and traits, and especially environmental factors. Unlike previous efforts in using transcriptomic datasets, this is now also done in large collaborative efforts, increasing robustness and value of the results. Methylation signatures were identified for smoking [351], alcohol consumption [352], low grade inflammation [353], lipids [354], body mass and the adverse outcomes of adiposity [355].

Epigenome-wide studies have identified methylation sites associated with liver enzymes and hepatic steatosis and also a peripheral blood DNA methylation signature of hepatic fat with a potential causal pathway for non-alcoholic fatty liver disease [356, 357]. Furthermore, a finite set of DNA methylation markers (13 CpGs) were identified that allow accurate inference of smoking habit, with comparable accuracy as plasma cotinine use, and smoking history from blood, which is useful in epidemiology and public health research as well as in medical and forensic applications [351].

A number of studies have focused on the relationship between diverse molecular layers and (biological) aging. An integrative cross-omics analysis of DNA methylation sites have identified multiple CpGs for T2D, glucose and insulin homeostasis, and further showed the differential methylation explains at least 16.9% of the association between obesity and insulin [96]. Multi-omics analysis using the Rotterdam Study epigenetics and transcriptomics data also showed that regulatory mechanisms affecting the expression of *IREB2* gene, such as DNA methylation, may explain the association between genetic variants in chromosome 15q25.1 and COPD, largely independent of smoking [336]. Likewise, a systematic analysis integrating GWAS, gene expression and DNA methylation data indicated multiple long non-coding RNAs associated with cardiometabolic disorders [358]. Besides, functional genomics by integrating GWAS data and various experimental studies using human iPS-derived neuronal progenitor cells and miR-142 knockout mice demonstrated the role of miR-142 in the pathogenesis of Alzheimer’s disease [359]. An independent study further identified that the clinical spectrum of early Alzheimer’s disease pathology is explained by different biological pathways, in particular, the endocytosis, clathrin/AP2 adaptor complex, and immune response pathways, that are independent of apolipoprotein E (*APOE*) [360].

For additional EJE references please see [361–373].

## Nutrition and lifestyle epidemiology

### Overall aim and focus areas

The main aim of the Nutrition & Lifestyle research line is to evaluate how nutritional factors and lifestyle behaviors, such as diet, physical activity, smoking, alcohol consumption and obesity, are associated with population health across the life-course. In our group we study determinants of lifestyle factors and its trajectories, associations of lifestyle with health and disease, underlying mechanisms of these associations (e.g., epigenetics, microbiome composition, inflammatory markers), and how these may differ for different groups of people (e.g., by other environmental factors, age, or genetic make-up).

### Key methods and data collection

Within the Rotterdam Study, data on several nutritional and lifestyle factors have been collected. Various behaviors, such as alcohol consumption, coffee intake, and smoking are measured with questionnaires. In older participants, appetite is assessed using the Council on Nutrition appetite questionnaire (CNAQ) [374]. For overall dietary intake, we use very comprehensive food-frequency questionnaires (FFQ) [44], from which data is available on food intake, nutrient intake, and various dietary patterns and scores.

In addition to self-reported dietary data, several nutritional biomarkers have been assessed, such as serum vitamin D, fatty acids, and vitamin B12. Nutritional status and adiposity are also determined by anthropometric measurements, but also with Dual-energy X-ray absorptiometry (DXA), to distinguish total-body and area-specific body fat, lean and bone mass.

Objective measurements of activity are performed with triaxial accelerometers (GeneActiv). Rotterdam Study participants are asked to wear these for 7 consecutive days and nights, from which we extracted information on for example time spent in light to vigorous activity, sitting, or sleeping [375]. Additionally, we use questionnaires, currently the International Physical Activity Questionnaire (IPAQ) [376], to measure various domains of activity, sedentary behavior and specific sports.

Finally, in addition to these lifestyle factors, we are also estimating exposure of Rotterdam Study participants to air pollution; to ambient particulate matter (PM2.5, PM2.5 absorbance and PM10) and nitrogen oxides (NO2 and NOx). In this ongoing effort we use the Land Use Regression (LUR) models developed within the European Study of Cohorts for Air Pollution Effects (ESCAPE) project [377].

### Main findings in the last 3 years

Overall, lifestyle of the Rotterdam Study participants is suboptimal. Although physical activity levels are generally high, approximately two-thirds of participants were overweight or obese at baseline, about a quarter of the population smoked, and adherence to dietary guidelines was low [44, 378, 379].

These lifestyle factors are major risk factors for premature mortality [44, 380, 381] and several diseases. In the Rotterdam Study, we observed for example that more physical activity is associated with lower mortality [380], lower cardiovascular disease risk [382], and with better quality of life [383]. Independent of physical activity, is better diet quality associated with a lower risk several diseases, such as age-related macular degeneration [282], non-alcoholic fatty liver disease [384], colorectal cancer, chronic obstructive pulmonary disease, and stroke [44]; with better bone health [385], brain health [386]; and with better overall health, as assessed with a frailty index [387, 388].

More specifically, in several studies we focused on the role of nutrition in the development of developing type 2 diabetes. We observed that a more plant-based diet and a lower intake of animal protein are associated with lower insulin resistance over time and lower risk of diabetes, independent of other lifestyle and dietary factors [205, 389]. In other analyses we studied several pathways that may link nutrition or physical activity to diabetes risk and metabolic health, such as adiposity [390], inflammatory pathways [391, 199] or DNA methylation [392–395].

We also identified several determinants of lifestyle. In the Rotterdam Study, diet, physical activity and insulin resistance differ by sociodemographic factors such as education status [378, 396], and interestingly, also by season [397–399]. In international consortia projects, we identified several genetic determinants of dietary intake [400]. We also studied interactions of diet or other lifestyle factors with genetic predisposition to disease in influencing disease risk [226, 395, 400–402]. Although evidence remains inconsistent, overall we found no evidence to tailor dietary recommendations to genetic risk profiles for prevention of diabetes [400] or other diseases.

Finally, as lifestyle behaviors are often highly correlated [44, 403], we also study these factors in combination. We constructed a lifestyle score, including smoking, alcohol, diet quality, weight status, and physical activity [379] and observed that a better overall lifestyle was associated with better overall health, not driven by any specific lifestyle factor, confirming that a healthy lifestyle, i.e., a healthy diet, not smoking, no or low alcohol consumption, sufficient physical activity and a healthy weight, are all important in maintaining or improving health.

### Future perspectives

As lifestyle and its related health conditions change over time, we aim to collect more repeated and detailed measurements, for example for objective measures activity, diet, and detailed body composition. These data will allow us to study trajectories of lifestyle factors in time, determinants of these changes, and how these relate to changes in subclinical markers of health of disease. This will also allow us to better study the role of lifestyle among those with lifestyle-dependent conditions such as obesity and diabetes, identifying patterns and markers in lifestyle that could help not only in primary prevention but also in management and reversal of these diseases. Overall, this will help us to better understand how lifestyle and environmental factors relate to health and disease over time and for different groups of people.

For additional EJE references please see [404–433].

## Pharmacoepidemiology

### Overall aim and focus areas

Pharmacoepidemiology is a branch of clinical epidemiology in which the drug is the determinant of interest, either as a cure [efficacy and effectiveness] or as a cause of disease [drug safety].The focus in this research line is on drug effects, irrespective of the question whether these are beneficial or adverse. Consequently, environmental and biological determinants for drug effects are studied, notably genetic and epigenetic ones, as well as other biomarkers if available. Hereto, we focus on a large variety of biological and disease endpoints which are gathered in the Rotterdam Study to make use of the wealth of detailed information from these databases.

### Key methods and data collection

The main data source consists of the complete medication records of almost all participants in the Rotterdam Study as of January 1st 1991 from all pharmacies serving the Ommoord region with details on the product and international non-proprietary name, number of filled tablets/capsules, strength, prescribed daily dose and duration of use. As the pharmacy data do not include over-the-counter (OTC) drugs, all participants receive a complete medication review on each of the regular interview rounds. In this way, it is also possible to study adherence [compliance] to pharmacotherapy. This combination of pharmacy and interview data is quite unique in pharmacoepidemiology as the large majority of healthcare databases, as well as population studies miss either medication filling data or interview data. For a random sample of 2000 participants of the Rotterdam Study, blood samples are available to assess drug levels in users. This can be employed for pharmacokinetic/pharmacodynamic modeling of drug effects. An important focus is on pharmacogenetic modeling of drug effects, thanks to the availability of genome-wide association data, exome sequencing, DNA methylation, and –omic data. An interesting endpoint is the daily dose of drugs which are titrated by reference to clinical response, such as bradycardia to beta-blockers or hypoglycemia to glucose-lowering medicines. Because the prescribed daily dose is known on each day of the follow-up, we can study genetic determinants for dose response.

### Main findings in the last 3 years

During the past 3 years, pharmaco-epidemiologic studies were performed on the effects of several medicines with outcome data from the Rotterdam Study, notably: statins; other cardiovascular drugs such as thiazides, beta-blockers, ACE-inhibitors, and hypoglycemics such as metformin, insulin and sulfonylureas; antidepressants; benzodiazepines; and proton pump inhibitors.

Statins may cause myopathy and a GWAs demonstrated several SNPs associated with a higher risk of myopathy in an international consortium study [434]. Also, the HDL-response to statins seems to have a genetic basis [435]. Furthermore, studies were published on the association between statins and diabetes of which the risk was increased in users [436], and carotid plaque composition which was associated with a more calcified stabilized form while on statins [135]. On the other hand, anticoagulants increased the risk of intraplaque hemorrhage in carotid arteries [137]. In a comparison of the American ACC/AHA and European ESC guidelines for prescribing statins, considerable differences were found [437]. As for other cardiovascular drugs, GWAs were published on the pharmacogenetic determinants of the effects of ACE-inhibitors on serum potassium [438] and the risk of intolerance [439], and of the effects of thiazide diuretics on serum potassium [438], and the QT-interval [440]. Thiazides also proved to be associated with a lower serum level of magnesium [441]. In a meta-analysis of fall risk in elderly, non-selective beta-blockers were associated with an increased risk [442]. This was also the case with benzodiazepines of which the fall risk was associated with certain CYP2C9 genotypes [443]. Interesting results came from a meta-analysis in which several published candidate genes for the pharmacokinetics of metformin were tested for their hypoglycemic response in users. These candidate transporter gene variants had little contribution to variability in glycemic response to metformin in type 2 diabetes [444]. However, the glucose transporter gene SLC2A2 was an exception [445]. One other GWAs with sulfonylurea hypoglycemics and their effect on QT, JT, and QRS-intervals showed 8 novel loci with significant association after Bonferroni correction [446].

Furthermore, several studies were performed on the effects of antidepressants [447–454]. SSRI antidepressants were associated with a better subjective sleep [448] but with an increased risk of intracerebral microbleeds [454], decreased insulin secretion [450], and with a lower heart-rate variability [453] but at variance with earlier studies not with a decrease of bone mineral density [449]. Interaction of variants in *BRE* and *UBE2E2* with tricyclic antidepressants were identified in relation to RR intervals while among Hispanic/Latinos, variants in *TGFBR3* modified the relation between TCAs and QT intervals [452].

Also, we studied resistance to antibiotics [455–457]. Use of certain food constituents such as chicken, pork and cheese were associated with higher resistance to certain antibiotics [456], such as ciprofloxacin [455]. Antibiotics were associated with a prolonged disturbing effect on the microbiota [457].

### Future perspectives

The Pharmacoepidemiology Unit follows a determinant-focused research line. First, we investigate determinants for pharmacokinetic and pharmacodynamics effects of drugs on a population-based scale. These determinants are often genetic and include hypothesis-generating GWAs and hypothesis-testing candidate studies but include epigenetic- and omic studies. Second, we take advantage of the wealth of very detailed information as gathered in the Rotterdam Study by the outcome-focused research lines such as cardiovascular, locomotor and neurological epidemiology. This is possible because pharmacotherapy covers basically all disease outcomes and the sophisticated endpoints from other groups facilitate very subtle analyses of drug effects.

For additional EJE references please see [458–462].

## Population imaging in epidemiology

### Overall aim and focus area

Population Imaging entails the large-scale acquisition of medical images in controlled population-based cohorts, allowing to investigate structural and functional changes in the human body that may indicate early disease, can be used to identify persons at risk of developing disease, or may aid in disease prediction. An important focus for the population imaging research line within The Rotterdam Study relies on using imaging data to study etiology and prediction of neurodegenerative and cardiovascular diseases (including cerebrovascular diseases).

### Key methods and data collection

Population imaging within the Rotterdam Study currently comprises brain MR imaging (multiple time points; more than 12,000 scans in over 8000 individuals), CT-assessed arterial calcification (2500 persons, follow-up imaging currently in progress), carotid MR imaging (over 1500 persons) and musculoskeletal imaging (knee MRI in over 800 subjects). Since 2018, we are also performing brain amyloid PET CT (with a florbetaben tracer) in 700 Rotterdam Study participants. We apply automated computer algorithms to process all imaging data to extract relevant imaging features (e.g. volumetric assessments, but also more advanced measures such as white matter tractography or structure shape on brain MRI scans; or shear stress measurements on carotid MRI and calcification patterns on vascular CT). In 2020, we will start in a subcohort of 200 participants high-field brain MRI (7 T) to study cerebral small vessels in more detail.

### Main results in the last 3 years

Normal brain aging still only sparsely understood, though it is an essential background to compare several age-related diseases against. We have written in the past 3 years several landmark papers which provide basic insight into structural and functional brain aging in the general population [463–466].

We have furthermore shown that white matter microstructure has added value over macrostructure in cognitive deterioration and that tract-specific regional deterioration of white matter in aging relates to cognitive performance [467], to risk of stroke [468] and to mortality [469]. This research is instrumental in changing our way of thinking of white matter as a ‘bulk substance’ into differentiated tracts with specialized functions in aging, and to understand that we need to study tract-specific changes in cognitive deterioriation and dementia. This work was awarded the Stroke Innovation Award.

With respect to cerebral small vessel disease, we have shown that in the general population, cerebral microbleed presence relates to risk of stroke and dementia [470, 471], further strengthening the view of microbleeds as a ‘missing link’ between vascular disease and neurodegeneration.

In our vascular calcification research, essential pioneering work was done on the importance of intracranial arteriosclerosis, which was established as one of the most important risk factors of first-ever stroke [472] and a contributor to dementia and migraine. This work has been an important cornerstone for fueling novel studies, including population-based studies and clinical studies in stroke patients (e.g. MR-CLEAN). We furthermore demonstrated that existing imaging-examinations (e.g. a coronary calcium CT-scan) contain a wealth of untapped information on other health parameters which yield additional information on the risk of cardiovascular disease [473].

With respect to carotid atherosclerosis and vulnerable plaque components on imaging, we have demonstrated recently that antithrombotic treatment relates to intraplaque hemorrhage [137] and that statin use seems to beneficially influence composition of carotid atherosclerosis by shifting towards more stable calcified plaque [135].

### Future perspectives

Imaging in population-based studies is becoming ever more important in studying determinants of disease and in disease prediction. Non-invasive imaging techniques, such as MRI, enable us to detect increasingly subtle and early pathologic changes in asymptomatic individuals, tremendously enlarging our power and sensitivity to study common diseases, like stroke and dementia. In the coming years, we expect particular progress to be made by exploring the interrelationship between structure and function of bodily tissues. Furthermore, advances in image processing, yielding quantification of more and new markers and data-driven artificial intelligence research techniques (machine learning, deep learning) will bring the field of population imaging forward. Also, combining imaging with other high-dimensional data such as genomics, proteomics and metabolomics, is highly promising in unravelling pathways of disease and better understand disease pathophysiology. Finally, we will focus in the next years even more on the clinical relevance and prognosis of the imaging markers assessed in our cohorts.

For additional EJE references please see [474–477].

## Emeriti principal investigators

The following persons are Principal Investigator Emeritus of the Rotterdam Study:

Frank van den Ouweland (PI Internal Medicine 1990–1992), Diederick Grobbee (PI Cardiovascular diseases 1990–1996), Albert Hofman (PI Neurological diseases 1990–1996, overall PI 1990–2016), Paulus de Jong (PI Ophthalmic diseases 1990–2005), Huibert Pols (PI Internal Medicine 1993–2006), Monique Breteler (PI Neurological diseases 1997–2010), Gabriel Krestin (PI Population Imaging 1998–2010), Johannes Vingerling (PI Ophthalmic diseases 2005–2010), Jacqueline Witteman (PI Cardiovascular diseases 1997–2011), Ernst Kuipers (PI Hepatogastrointestinal diseases 2007–2013), Harry Janssen (PI Hepatic diseases 2007–2013), Cornelia van Duijn (PI Genetic epidemiology 1990–2018), Henning Tiemeier (PI Psychiatric epidemiology 2002–2017), Oscar Franco (PI Cardiovascular diseases 2011–2018), Sarwa Darwish Murad (PI Hepatogastrointestinal diseases 2013–2018).
